# Simulating the Complex Cell Design of *Trypanosoma brucei* and Its Motility

**DOI:** 10.1371/journal.pcbi.1003967

**Published:** 2015-01-08

**Authors:** Davod Alizadehrad, Timothy Krüger, Markus Engstler, Holger Stark

**Affiliations:** 1 Institute of Theoretical Physics, Technische Universität Berlin, Berlin, Germany; 2 Department of Cell and Developmental Biology, Biocenter, University of Würzburg, Würzburg, Germany; University of Connecticut Health Center, United States of America

## Abstract

The flagellate *Trypanosoma brucei*, which causes the sleeping sickness when infecting a mammalian host, goes through an intricate life cycle. It has a rather complex propulsion mechanism and swims in diverse microenvironments. These continuously exert selective pressure, to which the trypanosome adjusts with its architecture and behavior. As a result, the trypanosome assumes a diversity of complex morphotypes during its life cycle. However, although cell biology has detailed form and function of most of them, experimental data on the dynamic behavior and development of most morphotypes is lacking. Here we show that simulation science can predict intermediate cell designs by conducting specific and controlled modifications of an accurate, nature-inspired cell model, which we developed using information from live cell analyses. The cell models account for several important characteristics of the real trypanosomal morphotypes, such as the geometry and elastic properties of the cell body, and their swimming mechanism using an eukaryotic flagellum. We introduce an elastic network model for the cell body, including bending rigidity and simulate swimming in a fluid environment, using the mesoscale simulation technique called multi-particle collision dynamics. The *in silico* trypanosome of the bloodstream form displays the characteristic *in vivo* rotational and translational motility pattern that is crucial for survival and virulence in the vertebrate host. Moreover, our model accurately simulates the trypanosome's tumbling and backward motion. We show that the distinctive course of the attached flagellum around the cell body is one important aspect to produce the observed swimming behavior in a viscous fluid, and also required to reach the maximal swimming velocity. Changing details of the flagellar attachment generates less efficient swimmers. We also simulate different morphotypes that occur during the parasite's development in the tsetse fly, and predict a flagellar course we have not been able to measure in experiments so far.

## Author Summary

*Typanosoma brucei* is a uni-cellular parasite that causes the sleeping sickness, a deadly disease for humans that also occurs in livestock. Injected into the mammalian host by the tsetse fly, the trypanosome travels through the blood stream, where it proliferates, and ultimately can be taken up again by a fly during a bloodmeal. In the tsetse fly, it continues its development with several morphological changes to the cell body plan. During its life cycle, the trypanosome meets different microenvironments, such as the mammalian's bloodstream and the tsetse fly's midgut, proventriculus, foregut, and salivary gland. The cell body of the trypanosome has the shape of a spindle along which an eukaryotic flagellum is attached. We have developed an accurate, *in silico* model trypanosome using information from live cell analyses. Performing computer simulations, we are able to reproduce all motility patterns of the blood-stream form in typical cell culture medium. Modifying the cell design, we show that the helical course of the flagellar attachment optimizes the trypanosome's swimming speed. We also design trypanosomal morphotypes that occur in the tsetse fly. Simulation science thereby provides an investigative tool to systematically explore the morphologcial diversity during the trypanosome's life cycle even beyond experimental capabilities.

## Introduction

The African trypanosome, *Trypanosoma brucei*, is the causative agent of the deadly nagana and sleeping sickness in livestock and humans, respectively, which still persist as a main public health and economic problem for inhabitants in sub-Saharan Africa [1]–[3]. Trypanosomes are protozoan parasites with an elongated cell body shaped like a spindle. Their swimming mechanism is rather complex and subject of current research [4]–[6]. Trypanosomes are propelled by a beating eukaryotic flagellum attached along the cell body [7]–[9], which causes the whole cell body to deform and thereby creates a complex motility pattern [4], [5], [10]. Cell propulsion is crucial for parasite survival, morphogenesis, cell division, and infection in the mammalian host [11]–[15] and also for the life cycle in the tsetse fly [16]. Trypanosomes replicate in the tsetse fly before being transmitted into the bloodstream of the mammalian host during the fly's bloodmeal [17]–[19]. Moving through the mammalian blood vessels, they infect the skin, spleen, liver, heart, eyes, and ultimately the central nervous system. This causes irritability, speech problems, sleep disruption, and ultimately leads to death within weeks to months [20]–[23].

The different physical microenvironments, which trypanosomes encounter in the tsetse fly and the mammalian host, continuously exert selective pressure, to which the cells adjust with a variability in their architecture and behavior. For example, trypanosomes swim faster in the crowded environment of blood compared to the purely viscous culture medium [4] and remove surface-bound antibodies using hydrodynamic drag forces [24], or they reverse their swimming direction under different realizations of confinement such as pillar arrays or collagen networks in order to avoid becoming trapped [4]. Changes in cell architecture are most prominent during the development in the tsetse fly. However, the adaptions the cells experience with changing environment on their way through the tsetse fly are not well characterized.

Here, the cell goes through periods of proliferation, shape transformations, and migration through the midgut, foregut, proboscis, and salivary glands [17]–[19], [25], [26]. In more detail, once ingested by the tsetse fly with an infected blood meal from the mammalian host, the bloodstream form (BSF) of the parasite transforms into the procyclic form (PC) within the midgut lumen [19], [26]. Some of these PC parasites cross the peritrophic membrane of the gut, which separates the blood meal from the midgut epithelium, and migrate to the proventriculus in the anterior midgut. During this phase, they increase their body length, go through the long mesocyclic form of trypomastigotes [19], [27], and ultimately assume the long, slender form of the epimastigote cell [19], [26]. The parasites again cross the peritrophic membrane and continue their journey towards the salivary glands while undergoing an asymmetric cell division [17], [19], [26]. In the salivary glands the resulting short epimastigote cells attach to the microvilli of epithelial cells using their flagella. They evolve into the final free metacyclic form and are injected with the fly's proboscis into the next mammalian host [19], [28]. Thus, the entire life cycle consists of several striking morphological modifications and takes around 20–30 days.

Although the development of the different cell forms in the tsetse fly has been analyzed [17]–[19], [26], [28], the specific morphological adaptions the cells undergo in response to changing environments on their way through the fly are not well characterized. For example, despite recent experimental advances, a detailed analysis of how the trypanosome interacts with the tsetse host during the different stages of its life cycle, remains challenging due to technical constraints and time consuming procedures of in vivo experiments [19]. Furthermore, reliable cell cultures of the trypanosomal morphotypes in the tsetse fly have not been established yet. Therefore, relatively little is known about underlying physical mechanisms governing the life cycle of the trypanosome.

Here the predictive power of our computational approach to complex cell design comes in. Based on earlier work [6], in this article we present accurate, nature-inspired cell models which we have developed using information from live cell analyses but also by interpolating between known forms. The cell models account for the detailed geometry, elastic properties, and motility of the real trypanosomal morphotypes including the attached flagellum. Our best established model accurately simulates all motility modes of the blood stream form including forward and backward swimming as well as tumbling. Specific and subtle modifications in details of the flagellar attachment reveal how the blood stream form is optimized for swimming. Modifications of the general body plan allow to simulate different morphotypes of the parasite in the tsetse host. The results demonstrate the true predictive power of our approach for the quantitative analysis of complex cell morphology and motility in a fluid environment. By constructing *in silico* morphotypes and investigating them in computer simulations, we are able to guide the interpretation of experiments or access complex cell designs, which cannot be studied systematically by experiments alone.

## Results/Discussion

### Realistic cell design and swimming mechanism

**Figure 1:**
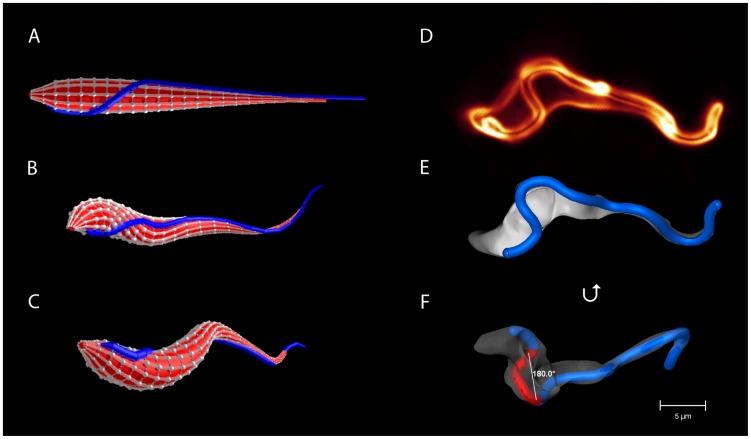
The model trypanosome and a real trypanosome. (a) Cell body of the model trypanosome without distortion. The elastic network made from vertices connected by springs defines the surface. The blue line connecting a series of vertices represents the flagellum with the helical half-turn. (b, c) Snapshots of the model trypanosome during simulated swimming motion. (d) 3d volume model of a live trypanosome with fluorescently labeled surface. (e) 3d surface model of the cell in (d), with the flagellum highlighted in blue. (f) The same surface model rotated about the horizontal, in order to get a better view on the left-handed half-turn of the flagellum indicated in red.

To simulate and analyze the trypanosome's swimming mechanism, we constructed a refined elastic network model of the trypanosome based on experimental data from advanced video microscopy. A full description of the model is presented in the Section *Materials and Methods*. We simulated the fluid environment with a mesoscale simulation technique for solving the Navier-Stokes equation called multi-particle collision dynamics (MPCD) [29]–[32]. The trypanosome has a spindle-shaped elongated cell body with tapered ends. The cell body is about 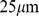 long, has a diameter of ca. 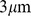 at the thickest part in the posterior section, and becomes thinner towards both ends, in particular, towards the long anterior end [4], [5]. Correspondingly, our model trypanosome has circular cross sections of varying diameter and the cell surface is defined by a mesh of points which are connected by harmonic springs spanning also in the cross-sectional planes [see Fig. 1(a)]. Together with additional bending rigidity, we obtain a cell body with precisely controllable flexibility. The actuating flagellum is attached to the cell body, which distorts in response to the bending wave running along the flagellum [see Fig. 1(b),(c)]. To adjust the course of the flagellum on the cell surface, we applied results from a detailed morphometric analysis using fluorescence microscopy [4]. An example for a cell body with fluorescently labeled surface is shown in Fig. 1(d) and in S1 Video. The rendered cell surface in Fig. 1(e) highlights the course of the attached flagellum and in Fig. 1(f) the whole cell is rotated by 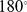 about the horizontal. From a careful inspection of such images the following flagellar course for the cell model evolved [see Fig. 1(a)]: The flagellum originates from the flagellar pocket at the posterior end of the cell, follows a small straight segment and then wraps around the cell body with a left-handed half-turn. Altogether this needs a length of ca. 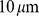 [4]. The flagellum then follows a straight path along the thinning anterior part of the cell body and protrudes freely at the anterior end. In the experimental images of Fig. 1(f) and S1 Video, the helical segment of the attached flagellum is marked red.

**Figure 2:**
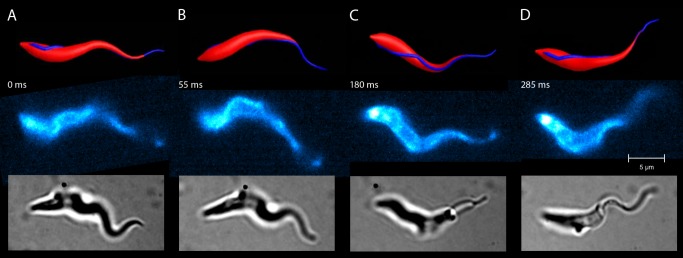
Comparison of the model and a real trypanosome during swimming motion (A–D). The swimming trajectory and dynamic shape of the simulated model trypanosome (top row) compares well with the forward swimming motion of the real trypanosome (middle and bottom row). Snapshots of the real trypanosome are taken at the indicated times from S4 Video. Fluorescently labelled surface (middle row) or untreated cells (bottom row) were recorded by high speed microscopy (200–500 frames per second). The cells, which exhibit similar speeds and rotational frequencies, show matching cell body conformations at all times over a swimming path of several cell lengths. These periodically repeating shape conformations are initiated by the bending wave passing along the flagellum and determine the trajectory of the swimming parasite.

We generate a sinusoidal bending wave along the flagellum, which runs from the thinner anterior tip of the cell body to the posterior end (tip-to-base beat). The bending amplitude decreases towards the posterior end to better match the shape changes of the real trypanosome. The wavelength 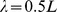, where 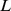 is the length of the cell body, is adjusted to the real system and the experimental wave frequency 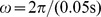 sets the relevant time scale [4]. The cell body distorts in response to the flagellar wave, which generates translational motion in the anterior direction opposite to the wave direction (see S2 Video). Due to the helical flagellar attachment, the cell body assumes an overall asymmetric chiral shape [see Fig. 1(b),(c)] typical of a real trypanosome [see Fig. 1(d),(e)]. To quantify the chiral shape, we determined the centerline of the model cell body, reaching from the posterior to the anterior end, and calculated the torsion averaged over the full length and over several time periods. As shown in the Section *Flagellar attachment optimizes swimming*, the non-zero mean torsion 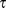 at the flagellar winding angle 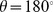 clearly indicates the overall chiral shape. Therefore, it rotates counterclockwise about the long axis of the cell, when viewed in the direction of motion (S2 Video). Typically, for a full turn 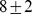 periods of the bending wave are needed in medium with the viscosity of blood [4]. Note, when the flagellar attachment is completely straight [winding angle 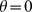], the mean torsion 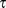 and hence the rotational speed are zero. In order to obtain a realistic motility pattern, the simulation model was empirically optimized to meet real life conditions. We adjusted the flexibility of the cell body accordingly by changing the elastic properties of the surface mesh and tuned the bending amplitude of the sinusoidal flagellar wave. Ultimately, this led to a realistic realization of the motility pattern of an African trypanosome as Fig. 2 and Videos S3 and S4 demonstrate.

**Figure 3:**
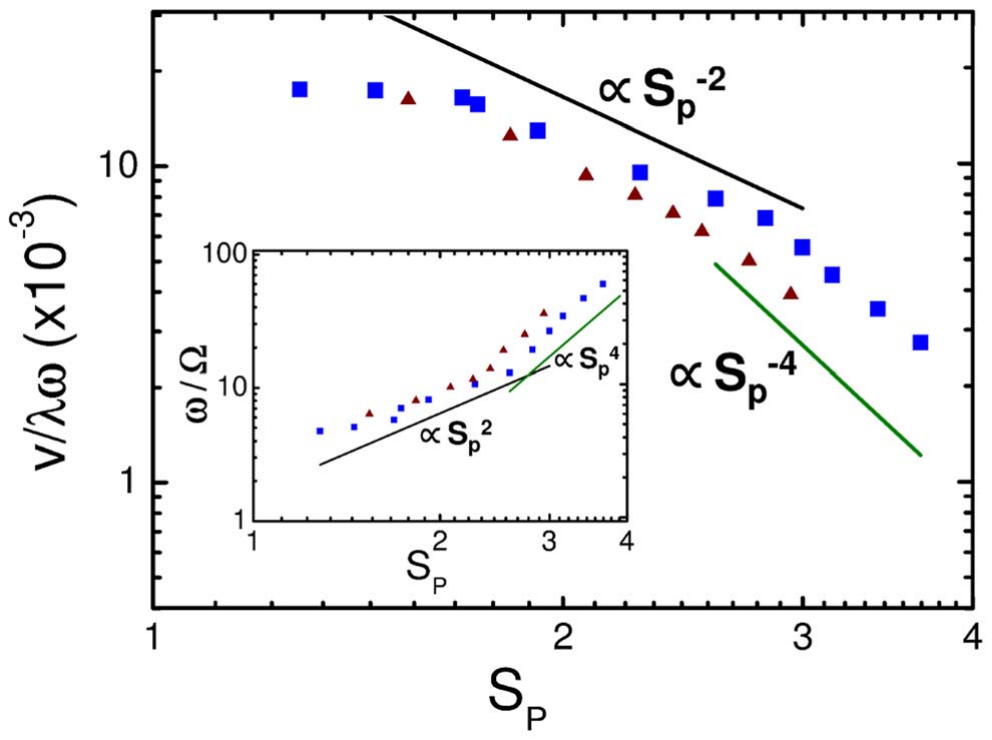
Swimming velocity versus sperm number. Rescaled swimming velocity 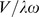 plotted versus sperm number 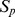 (double-logarithmic plot) for to two viscosity values 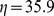 (squares) and 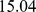 (triangles) given in MPCD units. Inset: Number of flagellar beats per full cell rotation 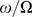 versus 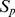. The solid lines indicate power laws in 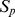.

The elongated model trypanosome can be regarded as a long slender body moving in a viscous fluid. For such an elastohydrodynamic system one expects a dimensionless parameter called the sperm number [33]–[35] to determine the complex propulsive dynamics of our model trypanosome [6]. The sperm number compares viscous to bending forces and is defined as 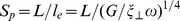, where 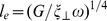 is the elastohydrodynamic penetration length, 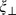 the perpendicular friction coefficient per unit length, 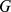 the bending rigidity of the cell body, and 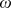 the angular frequency of the driving wave. In Fig. 3 we plot the rescaled swimming velocity 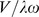 as a function of 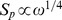 for two sets of shear viscosity 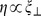 which approximately fall on the same master curve. Moreover, in the range from 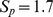 to 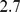, corresponding to an increase in frequency by a factor of 7, we approximately identify the scaling 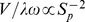 or 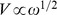 as predicted in [36] and in agreement with Ref. [6]. For larger values of 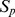 a second scaling regime occurs, which we attribute to the fact that the model trypanosome no longer swims in the quasi-static regime. The cell body rotates with an angular frequency 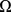. The number of flagellar beats per full rotation of the cell body, 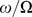, roughly scales as 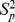 (inset of Fig. 3) in agreement with Ref. [6]. Beyond the quasi-static regime we find 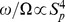. For 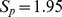, 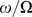 assumes the value 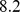, which matches the experimental value of 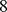 measured in medium with the viscosity of blood [4]. For this 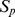 the resulting dynamics of the cell shape and the swimming pattern of the model trypanosome closely resembles the real swimming trypanosome as demonstrated in S3 Video.

### Tumbling dynamics and swimming backwards

We have designed a model trypanosome that very realistically reproduces the forward motion of the African trypanosome. We now test whether the model trypanosome can also show other motility patterns observed in experiments. Trypanosomes have the ability to reverse the direction of their flagellar bending wave and thereby swim backwards with a wave frequency 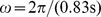, which is smaller compared to forward swimming by a factor of 0.6 [4]. Even waves travelling simultaneously from tip to base (forward swimming) and base to tip (backward swimming) are observed, especially in low viscosity fluids such as the standard cell culture medium [4]. With no predominant direction of the flagellar waves, this results in a tumbling motility pattern where the trypanosome constantly changes its swimming direction and typically produces no net translational movement at all. Tumbling is an important mechanism for cells to identify and swim along field gradients. It occurs in E. coli when the synchronized rotation of several flagella is perturbed [37] or when the two flagella of the algae Chlamydomonas beat out of synchrony [38]. Tumbling by two counterpropagating waves travelling along the trypanosome flagellum is an alternative strategy that we simulated with our model trypanosome. However, note that it is unclear if tumbling actually belongs to the in vivo behavior of the parasites, as they exhibit exclusively persistent directional swimming, when brought into surroundings corresponding to the confining situations in the bloodstream or in tissue [4]. In addition, there is no description of chemotactic abilities of trypanosomes so far.

**Figure 4:**
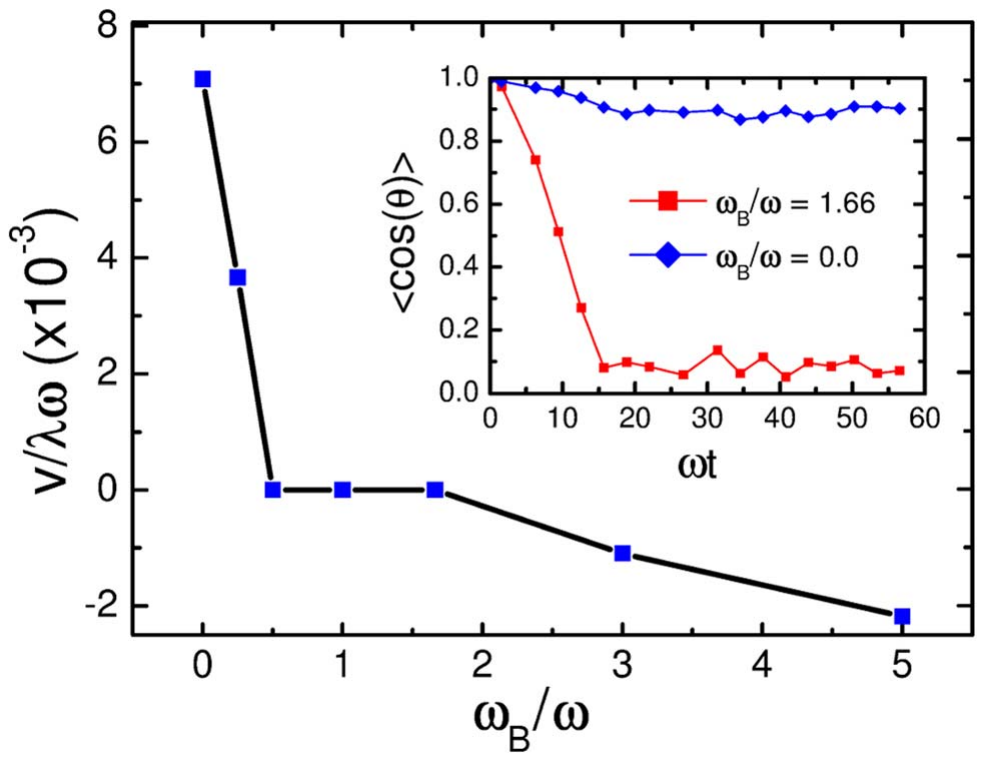
Swimming velocity versus the ratio of angular frequencies for counterpropagating flagellar waves. Rescaled swimming velocity 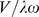 plotted versus the ratio 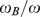 of angular frequencies for simultaneous base-to-tip (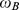) and tip-to-base (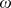) propagating flagellar waves. Inset: Orientational correlations of the end-to-end vector, 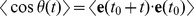, for two ratios of frequency.

We implemented bending waves travelling along the flagellum in both directions with increasing amplitude towards the tip. For both waves we chose the same wavelength 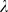. We kept the frequency 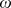 of the tip-to-base wave fixed and varied the frequency 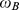 or velocity of the base-to-tip wave. Fig. 4 plots the reduced swimming velocity 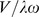 versus the ratio of both frequencies, 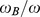. For 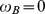, where we did not implement any base-to-tip wave, the trypanosome performs its standard motion in forward direction. At a ratio of 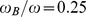, the swimming velocity is reduced to half the value and in the interval between 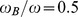 and 1.66 persistent swimming stops completely. For larger wave frequencies 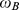 the swimming direction is even reversed as dictated by the base-to-tip wave. S5 Video shows both a real tumbling trypanosome in cell culture medium and the tumbling model trypanosome at 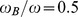. Both videos demonstrate with striking similarity the irregular motion and directional changes of a trypanosome. To quantify the directional persistence in the swimming motion, we determined the vector 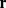 connecting both ends of the trypanosome. In the inset of Fig. 4 we plot the time-autocorrelation function for the orientation of the trypanosome, 
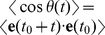
, where 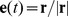 and 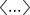 means average over reference time 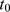 and several simulation runs [39]. Tumbling at 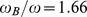 is indicated by a complete loss of orientational correlations after ca. three wave periods, whereas directional swimming at 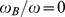 only shows a small decay, mostly due to the fact that the trypanosome does not swim on a perfectly regular helical trajectory, whereas small scale oscillations originate from periodic cell deformations. To conclude, our results demonstrate that disturbing the forward flagellar wave by a base-to-tip wave strongly affects the trypanosome motility pattern.

Trypanosomes perform sustained backward swimming with base-to-tip bending waves under conditions of confinement, i.e., in narrow spaces [4]. One can also force them to swim backward by inhibiting their forward motion. This is done when cells are depleted of the axonemal dynein motor protein DNAI1 by RNA interference against this protein. Cells missing a dynein outer arm can only produce base-to-tip flagellar waves [40] and thus constantly swim backwards in fluids with sufficiently large viscosities such as a culture medium with added methylcellulose solution which increases the viscosity to the value of blood viscosity [24]. Otherwise, the backward swimming is more erratic. We applied a sinusoidal bending wave to the model flagellum running from the posterior to the free anterior end. The wavelength 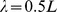 is the same as for the forward swimming mode but the wave frequency 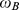 is reduced by a factor 0.6. We observe backward swimming and rotation about the long axis as in real cells. Remarkably, the swimming pattern was efficient only under conditions of confinement, just like the behavior of wild type trypanosomes. In S6 Video we compare the simulated cell moving backwards in a confining tube with a real trypanosome swimming persistently backwards after inhibition of forward motion by RNA interference.

In this article we concentrate on simulating the swimming trypanosome in a pure viscous fluid. In a viscoelastic environment such as blood or a collagen network and also in pillar arrays trypanosomes swim faster compared to the purely viscous culture medium since they use the suspended obstacles to drag themselves forward [4]. As already mentioned, in such confining environments trypanosomes do not tumble but swim persistently forward [4], although, they also reverse their swimming direction when they become trapped [4]. We currently investigate trypanosome swimming under the confinement of microchannels and in the presence of fixed and moving obstacles to mimic blood cells or the microstructure contained in viscoelastic fluids and clearly observe the enhanced swimming speed.

### Flagellar attachment optimizes swimming

**Figure 5:**
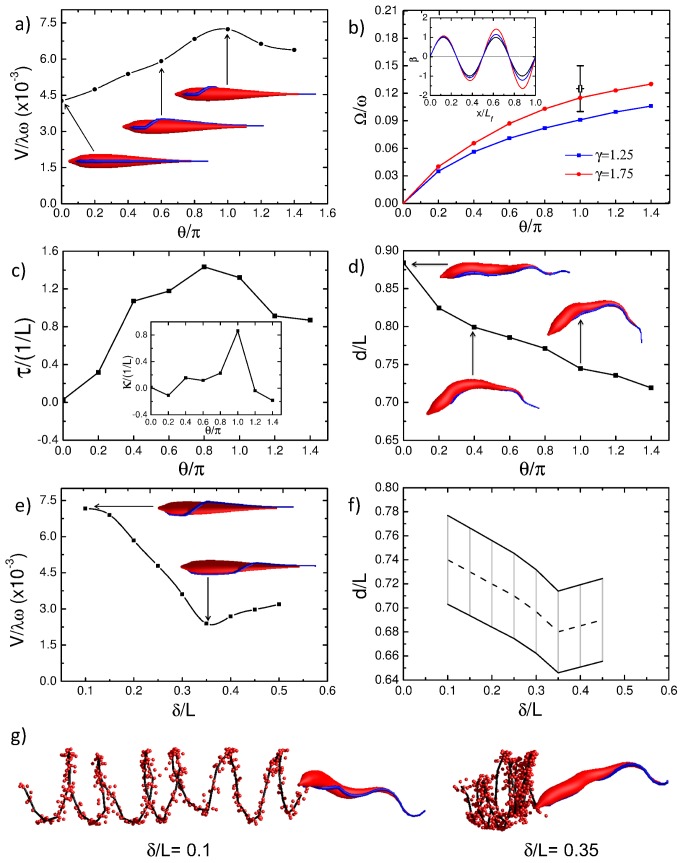
Swimming velocity, rotational frequency, torsion, and end-to-end distance for varying flagellar attachment. (a) Rescaled swimming velocity 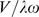 plotted versus the winding angle 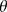 of the helically attached flagellum. The snapshots show the model trypanosomes before applying the bending wave. The flagellum winds counter-clockwise around the cell body up to the angle 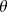. (b) Rescaled rotational frequency of the cell body, 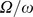, plotted versus the winding angle 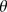. The inset shows how the amplitude of the imposed flagellar bending wave increases from the broader end of the cell body to the tip by a factor 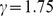 (red line), 1.25 (blue line), and 1 (black line). (c) Mean torsion 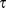 and mean curvature 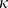 (inset) of the cell's centerline plotted versus 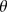. (d) Mean end-to-end distance of the cell body versus 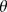. (e) Rescaled swimming velocity 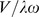 plotted versus reduced distance 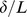 from the flagellar pocket, where the helical attachment begins. The snapshots show the model trypanosomes before deformation starts. The flagellum winds around the cell body always by 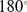. (f) Range of end-to-end distances 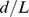 of the cell body during motion plotted versus 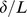. (g) Helical swimming trajectories of the posterior end indicated by red dots for 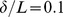 (left) and 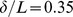 (right). Snapshots of model trypanosomes during swimming are illustrated.

We now use our model trypanosome to demonstrate how the flagellar attachment determined from video microscopy optimizes the motility pattern of the real trypanosome. In Fig. 5(a) we continuously tune the winding angle 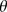 by which the flagellum wraps around the cell body from 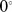 to well above the half-turn observed in the real cell. As before, the helical attachment begins after a short straight segment near to the flagellar pocket at the posterior end and then runs straight again towards the anterior end. Interestingly, the swimming speed plotted in Fig. 5(a) shows a clear maximum exactly at the half turn of the flagellar attachment. So the helical attachment seems to be optimized for the swimming speed. The helical attachment results in an overall chiral body shape which leads to rotational motion initiated by the flagellar wave [Fig. 5(b)]. The rotational motion then couples back to translational motion and enhances the swimming speed. A recent theoretical study of chiral microswimmers, driven by a torque, shows that the swimming speed is optimal, when the microswimmer has a bowlike shape rather than the form of a full screw such as the flagellum of an *E. coli* bacterium [41]. To quantify the shape of the model cell body, we determined its centerline and calculated from the local torsion and curvature values a mean torsion 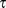 and curvature 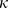 by averaging over the full cell length and several beating cycles of the flagellum. Details are given in the *Materials and methods* Section a). The results are plotted versus the winding angle 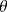 in Fig. 5(c), while Fig. 5(d) shows the cell's mean end-to-end distance 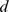 together with illustrative snapshots. The decreasing 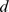 indicates the formation of a bow. In particular, for 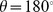 the mean curvature value 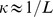 shows that the whole body is bent on an arc while the mean torsion, as a measure for the strength of chiral distortions, is close to its maximum value. Together with the results from Ref. [41], this gives some indication why the swimming speed in our case becomes maximal for a winding angle around 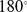. Fig. 5(b) shows how the rotational speed of the model trypanosome about the longitudinal axis continuously increases with the winding angle 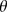, when the trypanosome becomes more chiral. Microscopic imaging reveals that the distortion of the real trypanosome at the anterior end is larger than at the posterior end. In our modeling of the trypanosome we take this into account by an increased bending flexibility of the anterior end but also by increasing the amplitude of the imposed flagellar bending wave. The inset of Fig. 5(b) illustrates the wave of the imposed bending angle for different growth factors 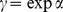, which is the ratio of the wave amplitudes at the anterior and posterior end, and 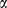 is explained in the *Materials and methods* Section b). By adjusting the growth factor to a sufficiently large value [two curves in Fig. 5(b)], we can match the rotational velocity 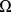 with the experimental value indicated by the error bar. This corresponds well with the approximate ratio of two inferred from microscopy images [4].

In Fig. 5(e) we demonstrate how the swimming speed 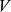 depends on the position of the flagellar half turn along the cell body, where 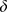 is the distance from the flagellar pocket. Our simulations show a reduction of swimming speed 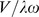 with increasing displacement 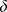 which clearly correlates with a reduction of the cell's end-to-end distance 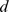. We plot the range of the oscillating 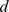 as a function of 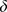 in Fig. 5(f). Cells with a larger end-to-end distance 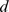 are more elongated. They experience less drag in the fluid and, therefore, move faster. We thus confirm an experimental observation that trypanosomes with a larger end-to-end distance swim faster [10]. When the helical turn of the flagellum is shifted towards the more flexible anterior end, the cell body bends more easily and swimming is no longer optimal. S7 Video and Fig. 5(g) impressively demonstrate the relevance of the optimized cell morphology for an effective cell motility. The cell with optimized parameters [Fig. 5(g), left and S7 Video, bottom] shows the typical rotational motion of a trypanosome about its longitudinal axis and efficient swimming along a helical trajectory. In contrast, the cell with shifted helical turn [Fig. 5(g), right and S7 Video, top] moves much slower and on a path with much smaller pitch.

Our trypanosome model allowed us to generate and investigate *in silico* mutants by varying the position and winding angle of the helical flagellar turn. We thereby revealed that for optimal swimming performance the flagellum has to be attached precisely as in real trypanosomes. All alternative designs produced less efficient microswimmers. Having such *in silico* mutants of the trypanosome available, has the advantage to study their motility and morphology during swimming in full detail. This is a significant advance compared to the difficulties inherent in experiments that analyze three-dimensional movements with two-dimensional video data [4] or the effort needed to record three-dimensional swimming trajectories by holographic microscopy [42].

### *In silico* morphotypes

We have demonstrated that we are able to reliably simulate all motility modes of the blood stream form of trypanosomes. We now proceed further to model other cell morphologies and simulate their swimming behavior. Whereas the blood stream form is well characterized, much less is known about the different morphotypes the trypanosome assumes in the tsetse fly [19], [26]. These morphotypes are difficult to analyze in *in vitro* experiments and, as yet, there are no established cell culture conditions that enable the correct development of the fly stages. Therefore, creating appropriate in silico morphotypes will be an important tool to analyze structure and motility of all possible forms of the trypanosome life cycle, in particular, in the tsetse fly.

When taking a blood meal on an infected mammalian host, the tsetse fly incorporates the stumpy form of the bloodstream trypanosome, which elongates and transforms into the procyclic form in the fly's midgut. The trypanosomes cell body and flagellum are continuously elongated further in the midgut to assume the mesocyclic form, which moves to the proventriculus and becomes the long slender epimastigote form, which divides asymmetrically to produce short epimastigotes. These finally transform further into the metacyclic form, which can infect the mammalian host again. During the development of the epimastigote form, the flagellar pocket moves to a more anterior position of the cell body [19].

**Figure 6:**
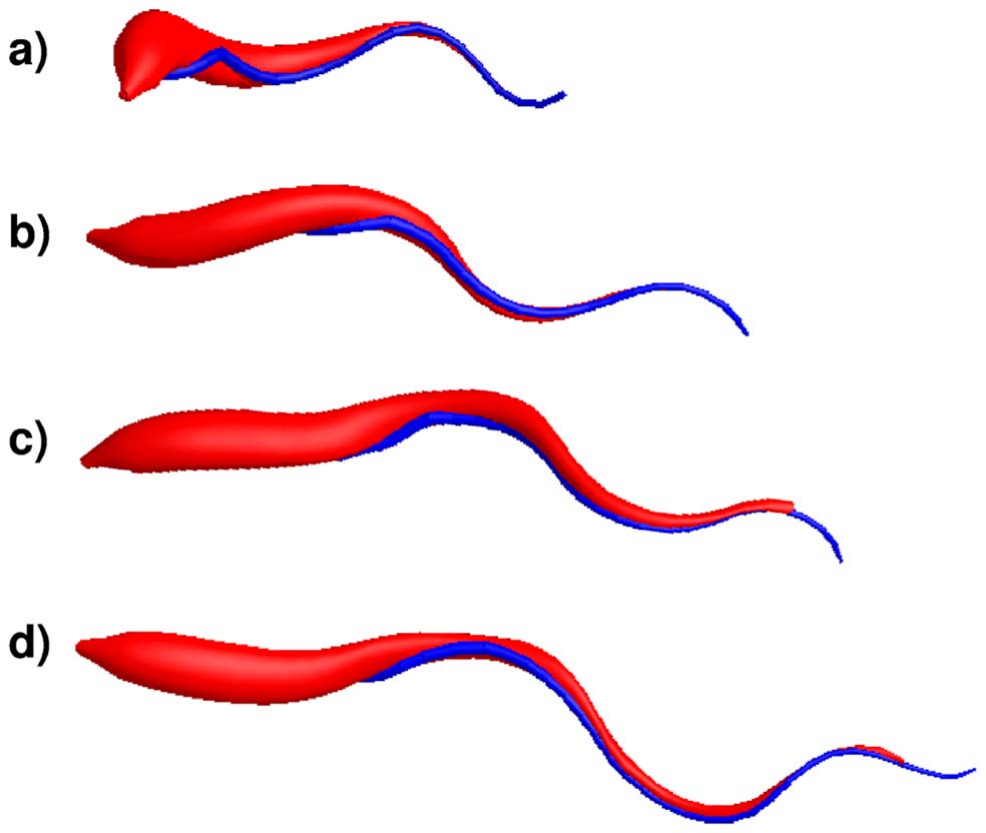
Snapshots of several *in silico* morphotypes during swimming. (a) The bloodstream form. (b) A possible intermediate morphotype in the tsetse fly, where the total cell length is increased by 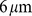 and the flagellum with the helical half-turn is displaced by 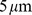 towards the anterior end. (c) A mesocyclic form with a total length of 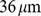. The flagellum starts at a distance of 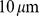 from the posterior end and the winding angle of the helical turn is tuned to 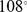. (d) A model that resembles an epimastigote form with a total length of 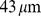, where the attachment of the flagellum is the same as for the mesocyclic form.

To model different morphotypes of the trypanosome, we tuned three morphological parameters: the position where the flagellum starts close to the posterior cell end, the cell length, and the length of the flagellum 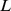, which grows with the elongating cell body. To avoid a bending instability of the thin anterior part of the cell body and to make the posterior end stiffer, we increased the bending stiffness by a factor of two. For the wavelength of the bending wave we chose 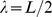, as before, and also kept the wave frequency constant. Fig. 6 shows snapshots of several *in silico* morphotypes, which we discuss in the following. Starting at the top, Fig. 6(a) illustrates the model of the bloodstream form used in the previous simulations. We then generate a possible intermediate morphotype in the tsetse fly [see Fig. 6(b)], where we increase the total cell length by 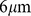 to 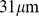 and displace the flagellum with its helical half-turn by 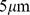 towards the anterior end. In Fig. 6(c) we illustrate an adjusted model for a mesocyclic form with a total length of 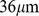, where the flagellum starts at a distance of 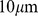 from the posterior end and the winding angle of the helical turn is tuned to 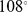 [see Fig. 6(c)], as explained below. Finally, elongating the cell model further towards the anterior end to a total length of 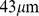 and keeping the same attachment of the flagellum [Fig. 6(d)], results in a model that resembles an epimastigote form.

**Figure 7:**
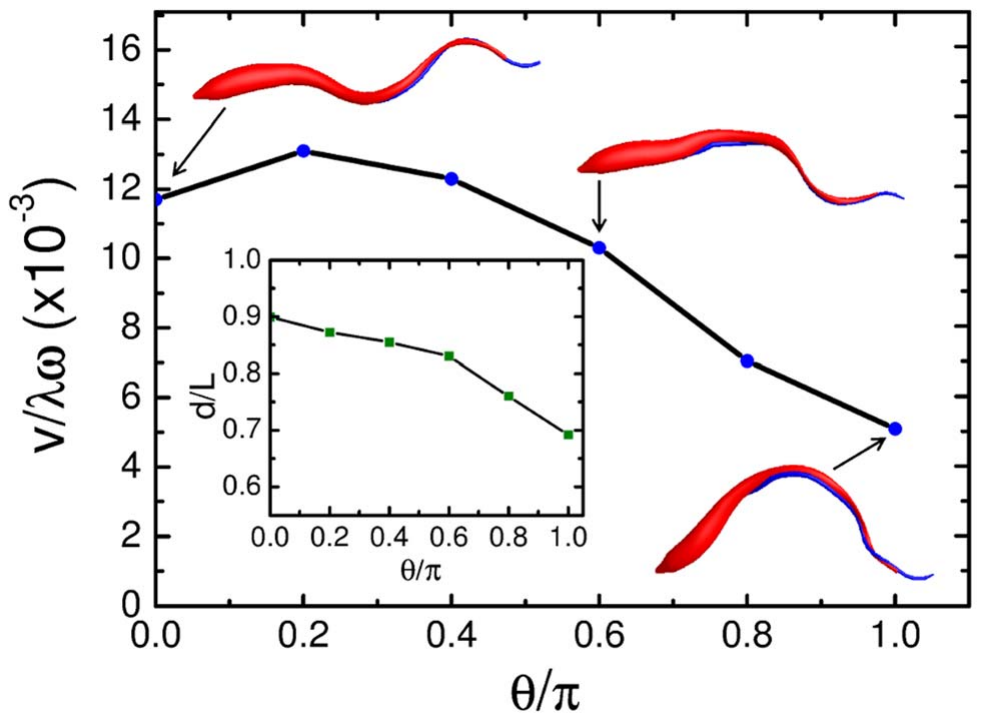
Swimming velocity versus flagellar winding angle for the mesocyclic form. Rescaled swimming velocity 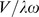 plotted versus winding angle 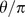. The snapshots show the model trypanosome for winding angles 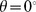, 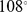, and 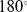. Inset: Rescaled end-to-end distance of the cell body, 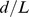, versus 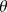.

To model the mesocyclic morphotype, we started with a helical half-turn of the flagellum and observed that the cell moved slower than the real mesocyclic form in experiments. We attributed this to the stronger bending of the simulated cell body compared to the real cell. Already in Figs. 5(c) and (d) we have demonstrated that stronger bending decreases the swimming velocity. We therefore decided to decrease the winding angle 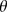 of the helical flagellar turn, which indeed lowered the bend of the cell body or increased the mean end-to-end distance 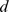, as the inset of Fig. 7 demonstrates. In parallel with the smaller bend, the cell becomes more straight and hence its hydrodynamic friction decreases. This, in turn, increases the swimming velocity (see Fig. 7). At angles around 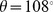 the flagellar bending wave produces the most realistic swimming pattern compared to the swimming mesocyclic trypanosome in experiments (see S8 Video), where speed and end-to-end distance of the model and the real trypanosome agree with each other. Also the rotational velocity of the cell body, which is lower than in the blood stream form due to the smaller helical turn, agrees well with experiments. In order to reduce the bend of the model trypanosome, other modifications of the cell body such as varying the stiffness of the cell or the amplitude of the flagellar wave were not successful. So we think that the reduced helical turn makes a solid prediction for the morphology of the mesocyclic cell. Last but not least, S9 Video presents our swimming *in silico* version that resembles an epimastigote form.

Experimental methods for analyzing in detail the morphology and swimming pattern of trypanosome forms inside the tsetse fly are currently being established in order to gather high-speed light microscopy and 3D morphometric date analogous to Ref. [4]. The technically demanding confirmation of our simulation results will demonstrate the predictive power of simulations based on accurate complex cell designs.

In conclusion, we have designed and constructed an *in silico* trypanosome using information from live cell analyses. We simulated and analyzed its swimming pattern with the help of the mesoscale simulation technique called multi-particle collision dynamics. The *in silico* bloodstream form accurately reproduces the characteristic forward swimming together with the rotational motion about the long axis, as well as the trypanosome's tumbling and backward motion. Specific modifications in the flagellar course around the cell body reveal that the flagellar attachment in the real cell maximizes the swimming performance. We then modified our cell model to simulate different morphotypes of the trypanosome in the tsetse fly. In particular, a comparison with a swimming mesocyclic trypanosome in experiments predicts a winding angle of 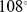 for the flagellar attachment and thereby makes a structural prediction for the cell morphology.

Our accurate cell modeling not only helps to explore design principles of real trypanosomes by performing specific modifications in the cell morphology, it also provides structural information which is not accessible with current experimental techniques. This demonstrates the predictive power of a sufficiently accurate *in silico* cell model. Similar to cell biology, we are able to generate *in silico* mutants of the trypanosome and thereby contribute new insights to cell morphogenesis during its life cycle. In future, we plan to explicitely explore the role of the attached flagellum during cell division.

## Materials and Methods

To simulate the swimming trypanosome, we construct an elastic network model for the cell body of the trypanosome and couple it to its fluid environment, which we treat with a mesoscale simulation technique called multi-particle collision dynamics (MPCD), an efficient particle-based solver of the Navier-Stokes equations [29]–[32] and by now well established in numerous studies (see, for example, [43]–[54]). A first implementation of our trypanosome model was reported in Ref. [6] together with a study of relevant system parameters. However, for being able to accurately simulate a swimming trypanosome and to reproduce many features of the real system, we had to extend and refine this model in close collaboration of theory and experiment using information provided by live cell analyses. In concrete terms, compared to Ref. [6] we corrected the flagellar attachment and extended the flagellum beyond the cell body at the anterior end. We differently quantified the decrease of the axial bending rigidity towards the anterior end and newly adjusted the involved elasticity constants of our model.

In the following, we review in detail the construction of the model trypanosome and the attached flagellum. Here, we shortly summarize how we used experimental information and empirical fitting to set up the model. Both the cell body shape and the path of the attached flagellum along the cell body are taken from experimental data. The shape of the bending wave running along the flagellum is not obvious due to the flagellar attachment to the cell body. We therefore decided to choose a sinusoidal variation of the prescribed curvature which is supported by the deformations of the very thin anterior end visible in experimental images (see, for example, lower row of Fig. 2). Also, Fig. 2 in Ref. [55], where the beating free flagellum of closely related Leishmania and Crithidia species are shown, nicely illustrates the approximate sinusoidal flagellar shape. To better match the deformation of the whole cell body [4], we introduce an amplitude of the flagellar bending wave that increases from the posterior to the anterior end and is described by an empirical fit parameter. Finally, parameters to describe the rigidity of the cell body against bending and twisting were assigned such that realistic cell deformations occur, which suffices to accurately describe the swimming behavior of a trypanosome. The parameters are not taken from experiments but in constructing the cell model we tried to mimic the cell cortex of the trypanosome.

### a) The model trypanosome

In the real cell, semi-flexible filaments called microtubules are attached to the cell membrane and run along the long axis of the cell body. They are linked to each other by proteins and therefore form a cortex that gives the trypanosome some stiffness, in particular, against bending [56]–[58]. The number of microtubules at a specific cross section of the cell body depends on the cell body diameter. It gradually reduces with the diameter towards both ends [56]–[58]. At the anterior, thinner end of the cell body, microtubules converge into a tightly closed tip and just a few microtubules reach the end, whereas at the broader posterior end many of them end at the same perimeter of the cell body, which creates an opening in the cortex [56]–[58]. Consequently the cell body becomes more flexible at the thinner part, particularly at the anterior end. Similar to red blood cells [59], [60], there are no filaments spanning across the cell so that it can deform easily. To implemented these characteristics, we constructed a model cell body for the African trypanosome on the basis of morphological data acquired from microscope images (Fig. 1 and Table 1). The cell body of the blood stream form has a total length 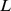 of about 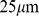, a relatively thick posterior end with a diameter of about 3 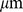, and a very thin anterior end [4], [5]. Accordingly, we constructed a model trypanosome with a spindle-like shape whose surface is represented by an elastic network of vertices. The size and shape of the model cell body is adaptable and hence we are able to simulate completely different morphotypes. This is demonstrated for the blood stream form (Fig. 1 and Videos S2, S3, and S4) and the mesocyclic and epimastigote forms in the tsetse fly (Fig. 6, Fig. 7, S8 Video, and S9 Video).

**Table 1:** The model cell body is constructed with a sequence of circular cross sections indexed by 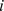.

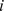	1	2	3	4	5	6	7	8	9	10	11	12
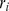	0.31	0.62	1.03	1.23	1.31	1.36	1.31	1.26	1.18	1.11	1.05	0.96
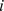	13	14	15	16	17	18	19	20	21	22	23	
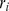	0.86	0.77	0.66	0.58	0.50	0.42	0.34	0.28	0.23	0.21	0.19	

Their radii 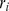, which we give in units of the MPCD length 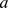, vary with 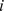 and were determined from microscope images. The distance of the circular cross sections in the simulations is determined by the equilibrium length 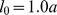 of springs connecting two vertices on neighboring cross sections. The resulting network model corresponds to the shape of an elongated trypanosome cell body of the blood stream form with a thicker posterior end and a very thin anterior end.

As shown in Fig. 1(a), the vertices are arranged in circles along the long axis of the cell body. The circles or cross sections of the cell body are defined by 10 equally spaced vertices and are indexed from 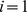 to 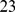 in the blood stream form starting at the posterior end. The diameters were determined from microscope images using the graphical software Plot Digitizer and are listed in Table 1. The vertices on the circle and from neighboring circles are connected by Hookean springs. Lines along the cell body also resist bending so that the complete potential energy of the elastic network becomes 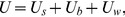
(1)where 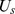 is the potential energy of the springs, 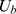 is the bending energy of lines of vertices running along the cell axis, and 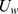 corresponds to the bending energy of the wave running along the flagellum. The harmonic spring potential 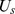 provides membrane elasticity similar to that of a trypanosome,
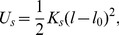
(2)where 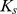 is the spring constant, 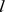 the actual distance of two vertices, and 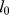 the equilibrium length of the springs. For springs connecting vertices of neighboring cross sectional circles, 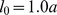, and the equilibrium lengths of the springs on a circle are 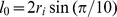 with the radii 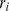 from Table 1. In order to stabilize the cylindrical shape of the cell body and to approximately ensure constant area of the cell surface and constant volume of the cell body, we introduce diagonal springs between opposing vertices on a circle. Furthermore, we model the bending rigidity of the cell body by applying the bending energy
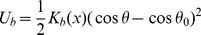
(3)to each line of vertices running from the posterior to the anterior end [see Figs. 1(a), (b), and (c)]. Here, 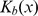 is the bending stiffness, and 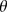 and 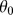 are the actual and the equilibrium angles between two bond vectors, respectively. The equilibrium values 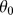 are adjusted to give the equilibrium shape of the trypanosome. Since the bending stiffness of the real cell body progressively reduces towards the thinner body part, which becomes very flexible at the anterior end, we choose the bending stiffness at a given point along the body long axis proportional to the local cross section 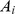,



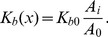
(4)Here, 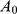 and 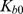 are the respective mean cross-sectional area and bending stiffness. This choice of 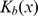 helps to mimic the microtubule system of the trypanosome. In the following we choose spring constant 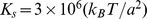 and bending rigidity 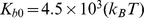, where 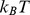 is thermal energy and 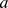 is the characteristic length of the MPCD method as explained below in part c). The parameters are chosen such that the cell is sufficiently stiff to guarantee a constant cell length (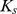) and thermal fluctuations of the cell body are negligible.

To quantify the cell body distortion, we determine the centerline of the cell body from the centers of its circular cross sections. For this centerline we determine the local values of curvature and torsion and average them over the whole centerline and several beating cycles of the flagellum. The curvature is a measure how strong a curve is bent in the osculating plane and torsion measures how strongly a curve moves out of the osculating plane. The mean torsion is therefore a measure for the chiral distortion of the cell body. Since the centerline is defined by discrete points 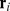, we use a discrete definition for curvature and torsion [61]. We define the normalized tangent vector at point 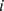 by 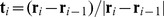 and the binormal by 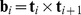. Then the local curvature and torsion become 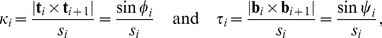
(5)where 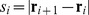. To average out undulations of the cell body induced by the flagellar wave, we assign to each local curvature a sign. For this, we define the normal vector 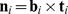 in the osculating plane. We start with defining a sign for the point 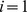. We keep this sign for the following 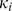 as long as 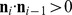. When 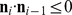 is encountered, the sign is reversed and the new sign is maintained as before as long as 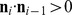 is satisfied. The sign of the torsion 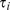 is given by the sign of 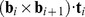. We then assign a positive 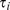 for a local left-handed screw. The mean curvature 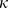 and torsion 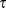 follow by averaging the local values 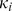 and 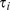 over the whole centerline and over several beating cycles of the flagellum.

### b) Modeling of the attached flagellum

The flagellum, which is composed of a classical 9 + 2 microtubule axoneme and a paraflagellar rod, emanates from the flagellar pocket close to the posterior end of the cell body and runs along the long axis towards the very thin anterior end [4], [5], [12]. It is connected to the cellular cortex by connecting proteins [7]–[9] and appears as a thicker rope attached to the cell body in electron microscopy pictures [5], [12], [62]. We model the flagellum as a line with an additional bending potential connecting already existing vertices on the cell surface as indicated in Fig. 1(a). High-resolution microscopy reveals that the flagellum even extends beyond the anterior part of the cell body [see Figs. 1(d)–(f)] [4], [5], [12]. Here we use 3–4 additional vertices to extend the flagellum beyond the tip [see Fig. 1(a)] with modified stretching and bending constants. We give the first additional vertex a bending rigidity of 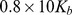, where 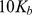 is the total bending constant of the cell body at the anterior end, and progressively reduce it by a factor of 0.8 for the following vertices. We choose the stretching constant equal to 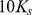. Because of the small bending rigidity of the free part of the flagellum, it can deform easily. Note the propulsive force of the trypanosome is significantly produced by the thinner part of the cell body and the free anterior piece.

The flagellum attached to the cell body is a typical eukaryotic flagellum driven by the collective motion of internal motors, which initiate a beating pattern along the flagellum. As S3 and S4 Videos demonstrate, there is a wave passing along the flagellum which distorts the whole cell body. To model this situation, we let pass a bending wave along the flagellum with constant frequency and wavelength, which travels from the free part to the thick posterior end of the cell body. To generate the bending wave, we use the bending energy 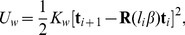
(6)where 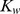 is the bending rigidity and 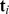 is a bond vector with length 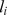 that connects vertices 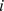 and 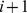 on the flagellum. We choose the bending rigidity 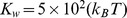 from an empirical optimization. The rotation matrix 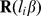 rotates 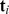 by an angle 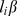 about the local normal of the cell body and thereby locally defines an equilibrium bending so that the groundstate of the flagellum is not straight. The local bending angle 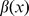 varies according to a sinusoidal travelling wave,
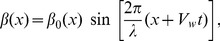
(7)where 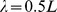 is the wavelength in units of the total cell length 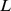, 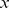 is the distance from the posterior end of the flagellum to its vertex 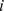, and 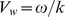 is the speed of the wave. It depends on the angular frequency 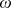 and the wave number 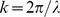. Microscopic imaging results show that the amplitude of the distortion wave along the cell body increases towards the anterior end [4]. Since we cannot model this just by the increased flexibility or reduced bending rigidity of the cell body towards the anterior end, we introduce a wave amplitude 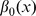 that increases from the broad posterior end of the cell body to the thin tip [see inset of Fig. 5(b)] according to



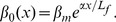
(8)Here 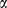 is a measure for the increase and 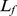 is the length of the flagellum.

### c) Modeling the surrounding fluid and coupling to the model trypanosome

To model the surrounding fluid and simulate the flow fields created by the swimming cell body, we use the simulation method of MPCD. The fluid is modeled by a finite number of pointlike particles of mass 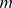 and with density 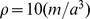, where 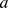 is the linear dimension of the collision cell to be introduced below. The point particles are distributed in a simulation box, typically with dimensions: 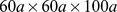. With 10 particles per collision cell, we simulate around 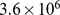 coarse-grained fluid particles. Their dynamics consists of alternating streaming and collision steps. In the streaming step, the particles move ballistically along their velocities during a given time interval 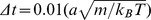, where 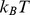 is the thermal energy. In the following collision step the simulation volume is divided into cubic cells of linear dimension 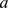 that contain 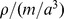 fluid particles. They interact with each other according to a specific collision rule (MPC-AT+a) adopted from the Anderson thermostat, which conserves linear but also angular momentum [32]. This procedure generates a solution of the Navier-Stokes equations. To ensure Galilean invariance, the cells for each collision step are generated with a random shift [63]. Both, the streaming and collision step contribute to the viscosity of the fluid, which can be tuned by density and 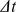. For 10 particles per collision cell and 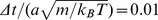, we obtain 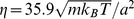, which we typically use in our simulations. A second value with 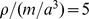 amounts to 
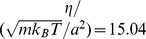
, which was also used for a few simulations. In the simulations all quantities are given in the respective MPCD units of length, time etc, introduced in the previous paragraphs.

The motion of the model trypanosome is coupled to the surronding fluid in serveral ways as explained in Ref. [6]. During the streaming step, the vertices of the cell model perform several molecular dynamics (MD) steps, where we update their positions and velocities using the velocity Verlet algorithm and forces, which result from the potential energy of the elastic network of the cell body [6]. To avoid numerical instablities, we choose a very small time interval for the MD step, 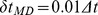. If a fluid particle penetrates into the cell body, it is reflected with a stochastic bounce back rule, which implements an approximate no-slip boundary condition on the cell surface. We distribute the momentum changes of the reflected fluid particles to the neighboring vertices of the cell body to conserve linear momentum. In addition, the vertices defining the cell body take part in the collision steps. This procedure, which combines both elastic and hydrodynamic forces acting on the cell surface, determines the deformation and dynamics of the cell body. We checked that the total force and torque acting on the trypanosome was zero, as it should be for a low-Reynolds number swimmer.

### d) Parallel computing

On a single CPU, simulating the swimming trypanosome on a path with length of one to two body lengths takes approximately three to six months. Therefore, to reduce the computation time to a reasonable value, we developed a scalable version of our computer code to be used for parallel computing. To distribute the global computational load to local processors (CPU cores), a domain decomposition method is introduced. The communication between neighboring processors uses a message passing interface (MPI) library including non-blocking communication. We developed an in-house code, which is written in C language. In a test for strong scaling, the resulting speed-up increased almost linearly with the number of processors which enables us to keep the simulation time below two weeks when 20 processors are used in parallel. To validate our parallel computer code, we simulated the diffusion of the passive cell body (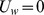) in the surrounding fluid and compared diffusion coefficients from parallel and single processor simulations with analytic results (S1 Fig.). The results nicely agree with each other.

### e) Cells and RNAi

Wildtype bloodstream form (BSF) *Trypanosoma brucei brucei*, strain 427, Molteno Institute Trypanozoon antigen type 1.6, was cultivated as described in [4]. Backward swimming trypanosomes were produced by RNAi against the dynein heavy chain (DNAI1) as described in [24]. RNAi was induced for 10 h. The pleomorphic strain *Trypanosoma brucei brucei* AnTat1.1 was cultured and tsetse flies were infected, maintained, and dissected as described in [26]. Flies were starved for at least 48 hours before being dissected. Dissection was performed 10 to 20 days after ingestion of the infectious meal. Tissues were then directly observed under the microscope or rapidly opened and flushed to resuspend parasites in culture medium or phosphate-buffered saline for further experiments.

### f) Surface staining

Live cells were surface-stained with 1 mM of AMCA-sulfo-NHS (Pierce, Rockford, IL) or Atto488-NHS (Atto-Tec, Siegen, Germany) for 10 min, immediately before each experiment. The incubation was carried out on ice and cells were kept in the dark. Unbound dye was removed by washing twice with ice-cold TDB at 2000xg for 90 s.

### g) Microscopy

Images were acquired with a fully automated fluorescence microscope iMIC (FEI), equipped with 100× (NA 1.4) and 60× (1.45 NA) objectives (Olympus), or a fully automated Leica DMI6000. Images were recorded with the CCD cameras sensicam.qe (PCO AG, Kelheim, Germany) or Leica DFC325fx. For high-speed light microscopy, a Phantom v9.1 camera (Vision Research, Wayne, NJ) was used and 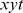-image series were acquired at 200–1000 frames per second (fps). For high-speed fluorescence microscopy, the sCMOS camera pco.edge (PCO) was used at frame rates of 200–400 fps. Cells were imaged in a two-dimensional setup of 10 mm height between a microscopic slide and a 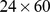 mm coverslip.

For 3D-modeling of fixed cells, 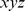 stacks were acquired in 100 nm steps. The cells were fixed in a final concentration of 4% w/v formaldehyde and 0.25% v/v glutaraldehyde in 0.1 M HEPES buffer over night at 4°C. The stacks were deconvolved using Huygens Essential software (SVI, Hilversum, Netherlands). 3D maximum intensity projection volume models were generated from these stacks, an edge detection filter (Sobel) was applied, and the model was false-colored in Amira (Visage Imaging, Berlin, Germany). Animations of 3D models and annotated Videos were produced with Amira or Imaris (Bitplane, Zürich, Switzerland). Flagella were traced in Amira. High speed videos of tumbling cells were manually annotated after single frame analysis in Amira. Arrows were included to follow every single wave crest travelling either from the anterior tip of the flagellum along the cell body to the thick posterior end (blue) or in the reverse direction from the posterior to the anterior end (yellow).

## Supporting Information

S1 FigMean-square displacement (MSD) of the center of mass of the passive cell body from parallel (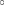) and single processor (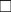) simulations. As expected for diffusive motion, 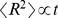. From the MSD we determine a diffusion coefficient 
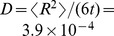
, which is in good agreement with the diffusion constant of a cylinder averaged over all orientations and moving in a viscous fluid of viscosity 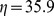: 
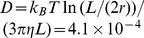
, where length 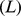 and radius 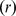 of the cylinder are equal to the length 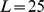 and mean radius 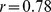 of the cell body, respectively, and all quantities are given in MPCD units.(TIF)

S1 VideoThree-dimensional volume models for three different snapshots of blood stream form trypanosomes, where the entire cell membrane and the flagellum are fluorescently labeled. The flagellum was traced manually and is shown in blue. The models are rotated in the video to show the typical course of the flagellum along the cell body. The animation is slowed for a view from the posterior end, in order to show the 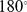 turn of part of the flagellum (in red) close to the posterior end of the cell body.(WMV)

S2 Video Simulated forward swimming motion of the trypanosome model for the bloodstream form. The flagellum (blue) is attached to the cell body along the full cell length. A small portion of the flagellum extends beyond the anterior end of the cell body (right). One third of the flagellum wraps in a half turn around the cell body. A sinusoidal bending wave propagates through the flagellum from the free anterior to the posterior end with decreasing amplitude and deforms the whole cell body. This generates both a translation swimming motion and a rotation of the model trypanosome.
(WMV)

S3 Video Comparison of the swimming trajectories of a simulated and a real bloodstream trypanosome. The upper video shows a persistently forward swimming cell in culture medium recorded at 500 fps. Both cells move at the same speed, have identical rotational frequencies, and show similar undulations of the cell body due to the bending wave propagating along the flagellum. Differences in the cell distortions are due to a slightly lower flexibility of the model trypanosome compared to the real cell.
(AVI)

S4 Video Comparison of the swimming trajectories of a simulated and a real bloodstream trypanosome in a medium with large viscosity. The upper and lower videos show persistently forward swimming cells in culture medium with 0.4 weight-% methylcellulose. This adjusts the fluid to the viscosity of blood, which is by a factor of ca. 5 larger than of pure cell culture medium. The upper video was recorded at 500 fps. The lower video of a trypanosome with a fluorescently labelled surface as in S2 Video was recorded at 200 fps.
(AVI)

S5 VideoSimulation of a tumbling trypanosome. The video on the left shows a bloodstream-form trypanosome recorded at 500 fps in culture medium. In such a low-viscosity fluid the trypanosomes typically exhibit flagellar waves running simultaneously from tip to base (indicated by blue arrows) and base to tip (indicated by yellow arrows) with varying frequencies. This results in a tumbling behavior with no or little directional motion. The video on the right shows tumbling simulated with the model trypanosome. The ratio of the flagellar wave frequencies for bending waves running from base to tip and from tip to base was 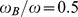, where we expect a zero swimming velocity.(WMV)

S6 Video Simulation of backward swimming. In the upper video a persistently backward swimming cell was recorded at 250 fps. In this cell the DNAI1 outer arm of dynein was depleted by RNA interference thus disabling tip-to-base flagellar waves. The cell exclusively generates flagellar waves from the posterior to the anterior end and in a high-viscosity medium moves persistently backward. The lower video shows a simulation of the model trypanosome with a base-to-tip flagellar wave. This generates persistent backward motion, for example, in a confining tube.
(WMV)

S7 Video Demonstration of optimized swimming performance. In the upper simulation the helical half turn of the attached flagellum starts in the middle of the model trypanosome, whereas in the lower video the half turn begins right at the posterior end as in the real bloodstream-form trypanosome. While the rotational speed is approximately constant, the swimming speed of the bloodstream form is maximal.
(WMV)

S8 VideoSimulation of a mesocyclic morphotype. The upper video shows a cell model, where the cell body was elongated and thinned. In addition, the position of the flagellar pocket was moved towards the anterior end and the helical turn was decreased to 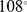. The resulting swimming pattern is very similar to the swimming mesocyclic form of the trypanosome isolated from the tsetse fly (lower video).(AVI)

S9 Video Simulation of an epimastigote-like form. Compared to the mesocyclic form, the model cell body was elongated and thinned further in order to simulate the epimastigote form. The position of the flagellar pocket and the helical turn of the flagellum are the same as in the optimized model for the mesocyclic form.
(AVI)

## References

[1] Maudlin I (2006) African trypanosomiasis. Ann Trop Med Parasitol 100: 679–701. 17227648 10.1179/136485906X112211

[2] Simarro PP, Jannin J, Cattand P (2008) Eliminating human african trypanosomiasis: Where do we stand and what comes next? PLoS Med 5: e55. 18303943 10.1371/journal.pmed.0050055 PMC2253612

[3] Simarro PP, Cecchi G, Franco JR, Paone M, Diarra A, et al (2012) Estimating and mapping the population at risk of sleeping sickness. PLoS Negl Trop Dis 6: e1859. 23145192 10.1371/journal.pntd.0001859 PMC3493382

[4] Heddergott N, Krüger T, Babu SB, Weia A, Stellamanns E, et al (2012) Trypanosome motion represents an adaptation to the crowded environment of the vertebrate bloodstream. PLoS Pathog 8: e1003023. 23166495 10.1371/journal.ppat.1003023 PMC3499580

[5] Rodríguez JA, Lopez MA, Thayer MC, Zhao Y, Oberholzer M, et al (2009) Propulsion of african trypanosomes is driven by bihelical waves with alternating chirality separated by kinks. Proc Natl Acad Sci USA 106: 19322–19327. 19880745 10.1073/pnas.0907001106 PMC2780783

[6] Babu SB, Stark H (2012) Modeling the locomotion of the african trypanosome using multi-particle collision dynamics. New J Phys 14: 085012.

[7] Koyfman AY, Schmid MF, Gheiratmand L, Fu CJ, Khant HA, et al (2011) Structure of trypanosoma brucei flagellum accounts for its bihelical motion. Proc Natl Acad Sci USA 108: 11105–11108. 21690369 10.1073/pnas.1103634108 PMC3131312

[8] Rocha GM, Teixeira DE, Miranda K, Weissmüller G, Bisch PM, et al (2010) Structural changes of the paraflagellar rod during flagellar beating in *Trypanosoma cruzi* . PLoS ONE 5: e11407. 20613980 10.1371/journal.pone.0011407 PMC2894934

[9] Hughes LC, Ralston KS, Hill KL, Zhou ZH (2012) Three-dimensional structure of the trypanosome flagellum suggests that the paraflagellar rod functions as a biomechanical spring. PLoS ONE 7: e25700. 22235240 10.1371/journal.pone.0025700 PMC3250385

[10] Uppaluri S, Nagler J, Stellamanns E, Heddergott N, Herminghaus S, et al (2011) Impact of microscopic motility on the swimming behavior of parasites: Straighter trypanosomes are more directional. PLoS Comput Biol 7: e1002058. 21698122 10.1371/journal.pcbi.1002058 PMC3116898

[11] Broadhead R, Dawe HR, Farr H, Griffiths S, Hart SR, et al (2006) Flagellar motility is required for the viability of the bloodstream trypanosome. Nature 440: 224–227. 16525475 10.1038/nature04541

[12] Ralston KS, Kabututu ZP, Melehani JH, Oberholzer M, Hill KL (2009) The trypanosoma brucei flagellum: Moving parasites in new directions. Annu Rev Microbiol 63: 335–362. 19575562 10.1146/annurev.micro.091208.073353 PMC3821760

[13] Ralston KS, Lerner AG, Diener DR, Hill KL (2006) Flagellar motility contributes to cytokinesis in trypanosoma brucei and is modulated by an evolutionarily conserved dynein regulatory system. Eukaryot Cell 5: 696–711. 16607017 10.1128/EC.5.4.696-711.2006 PMC1459671

[14] Kohl L, Robinson D, Bastin P (2003) Novel roles for the flagellum in cell morphogenesis and cytokinesis of trypanosomes. EMBO J 22: 5336–5346. 14532107 10.1093/emboj/cdg518 PMC213772

[15] Moreira-Leite FF, Sherwin T, Kohl L, Gull K (2001) A trypanosome structure involved in transmitting cytoplasmic information during cell division. Science 294: 610–612. 11641501 10.1126/science.1063775

[16] Rotureau B, Ooi CP, Huet D, Perrot S, Bastin P (2014) Forward motility is essential for trypanosome infection in the tsetse fly. Cell Microbiol 16: 425–433. 24134537 10.1111/cmi.12230

[17] Abbeele JVD, Claes Y, Bockstaele DV, Ray DL, Coosemans M (1999) Trypanosoma brucei spp. development in the tsetse fly: characterization of the post-mesocyclic stages in the foregut and proboscis. Parasitology 118: 469–478. 10363280 10.1017/s0031182099004217

[18] Vickerman K, Tetley L, Hendry KA, Turner CMR (1988) Biology of african trypanosomes in the tsetse fly. Biol Cell 64: 109–119. 3067793 10.1016/0248-4900(88)90070-6

[19] Sharma R, Gluenz E, Peacock L, Gibson W, Gull K, et al (2009) The heart of darkness: growth and form of trypanosoma brucei in the tsetse fly. Trends Parasitol 25: 517–524. 19747880 10.1016/j.pt.2009.08.001 PMC3770903

[20] Grab DJ, Kennedy PG (2008) Traversal of human and animal trypanosomes across the blood-brain barrier. J Neurovirol 14: 344–351. 19016378 10.1080/13550280802282934

[21] Kristensson K, Nygård M, Bertini G, Bentivoglio M (2010) African trypanosome infections of the nervous system: Parasite entry and effects on sleep and synaptic functions. Prog Neurobiol 91: 152–171. 19995590 10.1016/j.pneurobio.2009.12.001

[22] Kennedy PG (2004) Human african trypanosomiasis of the cns: current issues and challenges. J Clin Invest 113: 496–504. 14966556 10.1172/JCI21052 PMC338269

[23] Frevert U, Movila A, Nikolskaia OV, Raper J, Mackey ZB, et al (2012) Early invasion of brain parenchyma by african trypanosomes. PLoS ONE 7: e43913. 22952808 10.1371/journal.pone.0043913 PMC3432051

[24] Engstler M, Pfohl T, Herminghaus S, Boshart M, Wiegertjes G, et al (2007) Hydrodynamic flow-mediated protein sorting on the cell surface of trypanosomes. Cell 131: 505–515. 17981118 10.1016/j.cell.2007.08.046

[25] Fenn K, Matthews KR (2007) The cell biology of trypanosoma brucei differentiation. Curr Opin Microbiol 10: 539–546. 17997129 10.1016/j.mib.2007.09.014 PMC3902322

[26] Rotureau B, Subota I, Bastin P (2011) Molecular bases of cytoskeleton plasticity during the trypanosoma brucei parasite cycle. Cell Microbiol 13: 705–716. 21159115 10.1111/j.1462-5822.2010.01566.x

[27] Aksoy S, Gibson WC, Lehane MJ (2003) Interactions between tsetse and trypanosomes with implications for the control of trypanosomiasis. Academic Press, volume 53 of *Advances in Parasitology*. pp. 1–83. 10.1016/s0065-308x(03)53002-0 14587696

[28] Vickerman K (1985) Developmental cycles and biology of pathogenic trypanosomes. Br Med Bull 41: 105–114. 3928017 10.1093/oxfordjournals.bmb.a072036

[29] Malevanets A, Kapral R (1999) Mesoscopic model for solvent dynamics. J Chem Phys 110: 8605–8613.

[30] Malevanets A, Kapral R (2000) Solute molecular dynamics in a mesoscale solvent. J Chem Phys 112: 7260–7269.

[31] Padding JT, Louis AA (2006) Hydrodynamic interactions and brownian forces in colloidal suspensions: Coarse-graining over time and length scales. Phys Rev E 74: 031402. 10.1103/PhysRevE.74.031402 17025630

[32] Gompper G, Ihle T, Kroll D, Winkler R (2009) Multi-particle collision dynamics: A particle-based mesoscale simulation approach to the hydrodynamics of complex fluids. In: Holm C, Kremer K, editors, Advanced Computer Simulation Approaches for Soft Matter Sciences III, Springer Berlin Heidelberg, volume 221 of *Advances in Polymer Science*. pp. 1–87.

[33] Lowe CP (2003) Dynamics of filaments: modelling the dynamics of driven microfilaments. Philos Trans R Soc Lond B Biol Sci 358: 1543–1550. 14561345 10.1098/rstb.2003.1340 PMC1693247

[34] Dreyfus R, Baudry J, Roper ML, Fermigier M, Stone HA, et al (2005) Microscopic artificial swimmers. Nature 437: 862–865. 16208366 10.1038/nature04090

[35] Gauger E, Stark H (2006) Numerical study of a microscopic artificial swimmer. Phys Rev E 74: 021907. 10.1103/PhysRevE.74.021907 17025472

[36] Lauga E (2007) Floppy swimming: Viscous locomotion of actuated elastica. Phys Rev E 75: 041916. 10.1103/PhysRevE.75.041916 17500930

[37] Bray D (2001) Cell Movements: From Molecules to Motility. 2nd ed., Garland publishing, New York.

[38] Polin M, Tuval I, Drescher K, Gollub JP, Goldstein RE (2009) Chlamydomonas swims with two gears in a eukaryotic version of run-and-tumble locomotion. Science 325: 487–490. 19628868 10.1126/science.1172667

[39] Zaburdaev V, Uppaluri S, Pfohl T, Engstler M, Friedrich R, et al (2011) Langevin dynamics deciphers the motility pattern of swimming parasites. Phys Rev Lett 106: 208103. 21668266 10.1103/PhysRevLett.106.208103

[40] Branche C, Kohl L, Toutirais G, Buisson J, Cosson J, et al (2006) Conserved and specific functions of axoneme components in trypanosome motility. J Cell Sci 119: 3443–3455. 16882690 10.1242/jcs.03078

[41] Keaveny EE, Walker SW, Shelley MJ (2013) Optimization of chiral structures for microscale propulsion. Nano Lett 13: 531–537. 23317170 10.1021/nl3040477

[42] Weiße S, Heddergott N, Heydt M, Pflästerer D, Maier T, et al (2012) A quantitative 3d motility analysis of trypanosoma brucei by use of digital in-line holographic microscopy. PLoS ONE 7: e37296. 22629379 10.1371/journal.pone.0037296 PMC3358310

[43] Zöttl A, Stark H (2014) Hydrodynamics determines collective motion and phase behavior of active colloids in quasi-two-dimensional confinement. Phys Rev Lett 112: 118101. 24702421 10.1103/PhysRevLett.112.118101

[44] Tao YG, Kapral R (2010) Swimming upstream: self-propelled nanodimer motors in a flow. Soft Matter 6: 756–761.

[45] Huang MJ, Kapral R, Mikhailov AS, Chen HY (2012) Coarse-grain model for lipid bilayer self-assembly and dynamics: Multiparticle collision description of the solvent. J Chem Phys 137: 055101. 22894383 10.1063/1.4736414

[46] Downton MT, Stark H (2009) Beating kinematics of magnetically actuated cilia. Europhys Lett 85: 44002.

[47] de Buyl P, Kapral R (2013) Phoretic self-propulsion: a mesoscopic description of reaction dynamics that powers motion. Nanoscale 5: 1337–1344. 23282885 10.1039/c2nr33711h

[48] Prohm C, Gierlak M, Stark H (2012) Inertial microfluidics with multi-particle collision dynamics. Eur Phys J E 35: 80. 22926809 10.1140/epje/i2012-12080-3

[49] Babu S, Stark H (2011) Dynamics of semi-flexible tethered sheets. Eur Phys J E 34: 136. 10.1140/epje/i2011-11136-2 22197906

[50] Chelakkot R, Winkler RG, Gompper G (2012) Flow-induced helical coiling of semiflexible polymers in structured microchannels. Phys Rev Lett 109: 178101. 23215225 10.1103/PhysRevLett.109.178101

[51] Huang CC, Gompper G, Winkler RG (2013) Effect of hydrodynamic correlations on the dynamics of polymers in dilute solution. J Chem Phys 138: 144902. 24981544 10.1063/1.4799877

[52] Reid DAP, Hildenbrandt H, Padding JT, Hemelrijk CK (2012) Fluid dynamics of moving fish in a two-dimensional multiparticle collision dynamics model. Phys Rev E 85: 021901. 10.1103/PhysRevE.85.021901 22463238

[53] Winkler A, Virnau P, Binder K, Winkler RG, Gompper G (2013) Hydrodynamic mechanisms of spinodal decomposition in confined colloid-polymer mixtures: A multiparticle collision dynamics study. J Chem Phys 138: 054901. 23406143 10.1063/1.4789267

[54] McWhirter JL, Noguchi H, Gompper G (2009) Flow-induced clustering and alignment of vesicles and red blood cells in microcapillaries. Proc Natl Acad Sci USA 106: 6039–6043. 19369212 10.1073/pnas.0811484106 PMC2669370

[55] Gadelha C, Wickstead B, Gull K (2007) Flagellar and ciliary beating in trypanosome motility. Cell Motility and the Cytoskeleton 64: 629–643. 17549738 10.1002/cm.20210

[56] Hemphill A, Lawson D, Seebeck T (1991) The cytoskeletal architecture of trypanosoma brucei. J Parasitol 77: 603–612. 1865269

[57] Seeback T, Hemphill A, Lawson D (1990) The cytoskeleton of trypanosomes. Parasitol Today 6: 49–52. 15463295 10.1016/0169-4758(90)90069-g

[58] Gull K (1999) The cytoskeleton of trypanosomatid parasites. Annu Rev Microbiol 53: 629–655. 10547703 10.1146/annurev.micro.53.1.629

[59] Alizadehrad D, Imai Y, Nakaaki K, Ishikawa T, Yamaguchi T (2012) Quantification of red blood cell deformation at high-hematocrit blood flow in microvessels. J Biomech 45: 2684–2689. 22981440 10.1016/j.jbiomech.2012.08.026

[60] Alizadehrad D, Imai Y, Nakaaki K, Ishikawa T, Yamaguchi T (2012) Parallel simulation of cellular flow in microvessels using a particle method. Journal of Biomechanical Science and Engineering 7: 57–71.

[61] Sauer R (1970) Differenzengeometrie. Springer.

[62] Hill K (2003) Biology and mechanism of trypanosome cell motility. Eukaryot Cell 2: 200–208. 12684369 10.1128/EC.2.2.200-208.2003 PMC154846

[63] Ihle T, Kroll DM (2001) Stochastic rotation dynamics: A Galilean-invariant mesoscopic model for fluid flow. Phys Rev E 63: 020201. 10.1103/PhysRevE.63.020201 11308454

