# Supplementary figures and images for: Simulating the Complex Cell Design of Trypanosoma brucei and Its Motility
Source: PLoS Comput Biol. 2015 Jan 8;11(1):e1003967. doi: 10.1371/journal.pcbi.1003967 (PMC4288712; doi:10.1371/journal.pcbi.1003967)

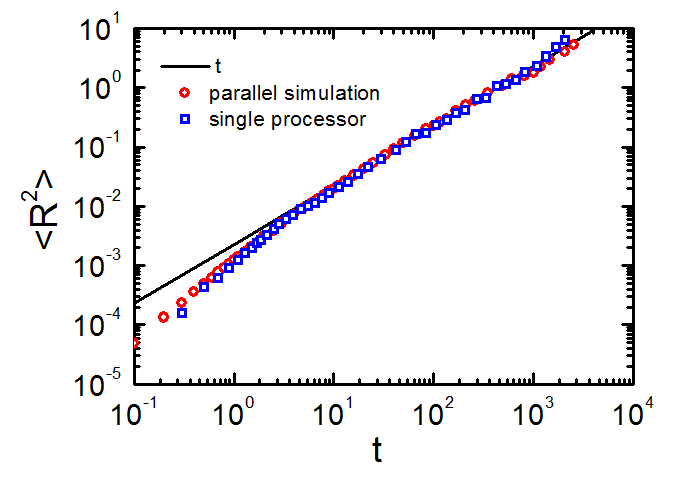

Supplement: S1 Fig — Mean-square displacement (MSD) of the center of mass of the passive cell body from parallel () and single processor () simulations. As expected for diffusive motion, . From the MSD we determine a diffusion coefficient , which is in good agreement with the diffusion constant of a cylinder averaged over all orientations and moving in a viscous fluid of viscosity : , where length and radius of the cylinder are equal to the length and mean radius of the cell body, respectively, and all quantities are given in MPCD units. (TIF) [file pcbi.1003967.s001.tif]
